# Measuring the impact of multiple sclerosis: Enhancing the measurement performance of the Multiple Sclerosis Impact Scale (MSIS-29) using Rasch Measurement Theory (RMT)

**DOI:** 10.1177/2055217317725917

**Published:** 2017-08-15

**Authors:** Sophie Cleanthous, Stefan Cano, Elizabeth Kinter, Patrick Marquis, Jennifer Petrillo, Xiaojun You, Craig Wakeford, Guido Sabatella

**Affiliations:** Modus Outcomes, UK; Modus Outcomes, UK; Biogen, USA; Modus Outcomes, USA; Biogen, USA; Biogen, USA; Biogen, USA; Biogen, USA

**Keywords:** Multiple sclerosis, clinical trials, psychometrics, Rasch Measurement Theory, MSIS-29, post-hoc analysis

## Abstract

**Background:**

Study objectives were to evaluate the Multiple Sclerosis Impact Scale (MSIS-29) and explore an optimized scoring structure based on empirical post-hoc analyses of data from the Phase III ADVANCE clinical trial.

**Methods:**

ADVANCE MSIS-29 data from six time-points were analyzed in a sample of patients with relapsing–remitting multiple sclerosis (RRMS). Rasch Measurement Theory (RMT) analysis was undertaken to examine three broad areas: sample-to-scale targeting, measurement scale properties, and sample measurement validity. Interpretation of results led to an alternative MSIS-29 scoring structure, further evaluated alongside responsiveness of the original and revised scales at Week 48.

**Results:**

RMT analysis provided mixed evidence for Physical and Psychological Impact scales that were sub-optimally targeted at the lower functioning end of the scales. Their conceptual basis could also stand to improve based on item fit results. The revised MSIS-29 rescored scales improved but did not resolve the measurement scale properties and targeting of the MSIS-29. In two out of three revised scales, responsiveness analysis indicated strengthened ability to detect change.

**Conclusion:**

The revised MSIS-29 provides an initial evidence-based improved patient-reported outcome (PRO) instrument for evaluating the impact of MS. Revised scoring improves conceptual clarity and interpretation of scores by refining scale structure to include Symptoms, Psychological Impact, and General Limitations.

**Clinical trial:**

ADVANCE (ClinicalTrials.gov identifier NCT00906399).

## Introduction

Growing interest in patient experiences of conditions like relapsing–remitting multiple sclerosis (RRMS) has led to increased interest in patient-reported outcomes (PROs).^1,2^ In addition to traditional endpoints, PROs are increasingly used in clinical trials to assess treatment effects from the patients’ perspective. The Multiple Sclerosis Impact Scale (MSIS-29) is a PRO developed in 2001 to assess multiple sclerosis (MS) according to two scales: Physical Impact and Psychological Impact.^3^ The MSIS-29 has been used in many different trials, including ADVANCE.^4,5^

The pivotal ADVANCE study compared the efficacy and safety of subcutaneous peginterferon 125 mcg beta-1a dosed every two and four weeks with placebo in patients with RRMS.^4,6^ Efficacy endpoints included adjusted annualized relapse rate (ARR) and confirmed disability progression (CDP), and MSIS-29 was an exploratory endpoint. Significant ARR and CDP treatment effects were demonstrated at Week 48 in both intervention groups compared with placebo.^4,6^ No significant treatment effects were demonstrated on the MSIS–29.

Within-scale changes from baseline indicated a significant improvement at Week 48 across all three treatment groups for Psychological Impact scores, and a worsening in Physical Impact scores (statistically significant in the placebo group only).^5^ A post-hoc analysis revealed that 12-week CDP was associated with a six-point worsening of the Physical Impact scores in the placebo group (*p* < 0.0001) relative to a 1.9-point worsening (*p* = 0.044) in the peginterferon beta-1a every-two-weeks group. Recent relapse (occurring within the past 29 days) was associated with a 10-point worsening of the Psychological Impact scores in the placebo group (*p* < 0.0001) relative to a 3.5-point worsening in the peginterferon beta-1a every-two-weeks group (*p* = 0.031).^5^

MSIS-29 and other PROs are valuable rating scales in evaluating treatment benefits from a patient’s perspective,^1,7,8^ measuring complex unobservable variables through self-reported questionnaires.^7,9^ A well-designed PRO should be reliable and valid, and care should be taken to ensure the total score of the scale appropriately reflects the patient experience.^1,7,10,11^ There are three main psychometric paradigms for developing and evaluating rating scales: Classical Test Theory (CTT),^12^ Rasch Measurement Theory (RMT),^13^ and Item Response Theory (IRT).^14^ A detailed comparison of these paradigms is presented elsewhere displaying the sophisticated and extensive analysis potential of the RMT.^1,8^

The MSIS-29 was developed in accordance with CTT criteria.^12^ Item questionnaires were generated through patient interviews (with primary progressive (PPMS), secondary progressive (SPMS), and RRMS patients), expert opinion, and literature review.^3^ Psychometric properties and responsiveness were evaluated in an independent sample^3^ and subsequently evaluated using CTT methods by several studies.^15–17^ However, in order to further assess the validity of the MSIS-29, it is important to validate the scale through an independent psychometric paradigm.

The RMT offers a mathematically testable model articulating a priori criteria used to verify measurement properties or to expose and explore anomalies of any rating scale empirically.^8,13,18^ An RMT of the MSIS-29 in a small sample (*n* = 92) of SPMS, RRMS, and PPMS patients was largely supportive of its measurement properties but revealed issues with its response scale, item fit, and coverage of patients with lower psychological impact.^19^ Two additional studies utilized portions of RMT analysis to assess item stability across participants with differential depression levels^16^ and patient and proxy responders,^20^ providing supporting evidence for MSIS-29. However, to date there have been no RMT analyses of the MSIS-29 conducted in a large sample of patients with RRMS.

The objective of this study was to evaluate measurement properties of the MSIS-29 within ADVANCE in accordance with RMT criteria. The RMT is superior to the CTT because it has interval rather than ordinal scoring, separation of item and person parameters, non-sample-dependent scale properties (e.g. reliability and validity), and the potential for individual-level measurement.^1,8^ The current RMT psychometric analysis enables extensive assessment of sample-to-scale targeting within RRMS patients whose levels of disability are potentially different from those of the patients’ used for the development and validation of the MSIS-29. Additionally, RMT allows for the optimization of the scoring structure and interpretation with the provision of interval-level scores.

## Methods

### ADVANCE study

ADVANCE was a two-year, randomized, double-blind, parallel-group, Phase 3 study, with a one-year placebo-controlled period comparing peginterferon beta-1a 125 mcg administered every two or four weeks versus placebo in patients with RRMS. Results from the study are described in detail elsewhere.^4^ ADVANCE recruited patients aged between 18 and 65 with a maximum 5.0 baseline Expanded Disability Status Scale (EDSS) score.^21^

Protocol for the ADVANCE study was approved by the institutional review board at each site, and the study was conducted according to International Conference on Harmonization Guidelines for Good Clinical Practice and the Declaration of Helsinki. Every patient provided written informed consent before entering the study.

### Materials

The MSIS-29 is a disease-specific PRO that measures the Physical and Psychological Impact of MS via two scales of 20 and 9 items each.^3,16^ Items are scored on five-point Likert-like scales, with higher scores indicating greater impact of MS on this domain.

### Data analysis

Data analysis followed three stages. First, psychometric evaluation of the MSIS-29 in line with RMT was performed. A review of these findings led to conceptual restructuring of the MSIS-29 measurement model, properties of which were evaluated using RMT at a second stage. Finally, the responsiveness of the original and rescored scales was examined. RUMM2030^22^ was used to conduct the RMT and IBM SPSS 21.0^23^ responsiveness analyses. Data from eight different time-points were stacked to increase sample size for the psychometric analysis.

### RMT analysis

RMT analysis compares observed data against the stringent criteria of the Rasch model with the broad aims described below.^8,24^

#### How adequate is the sample-to-scale targeting?

Sample-to-scale targeting concerns the match between the range of Physical or Psychological Impact measured by the MSIS-29 items, and the distribution of impact measured in the sample, subsequently influencing interpretation of all other RMT analyses. Person and item locations are plotted against the same metric, and their relative distributions are assessed.^25,26^

#### Do the response categories work as intended?

Greater changes in MSIS-29 scores signify more impact. It is therefore expected that the higher the impact of a responder, the higher the response category to be endorsed. Response thresholds are expected to be ordered in a successive manner along the measurement continuum.^8,27^ Thresholds represent the point at which the probability of endorsing two adjacent response categories is equal.

#### To what extent do the MSIS-29 items work together to define a single measurement construct?

RMT expects scale items to be cohesive and work well together clinically and statistically when summed up to a single total score.^8,27^ Using a rule of thumb, fit residual (residual = observed – expected score) estimates for each item should be within ± 2.5. Chi-square tests assess the difference between each item’s mean observed and expected scores within certain class intervals of the trait being measured. Item characteristic curves (ICCs) display this relationship graphically, providing context for interpreting the magnitude and pattern of numerical fit statistics.

#### To what extent does the response to one item bias the response to another?

RMT expects that items should not be dependent on or biased by each other so as to not artificially inflate reliability. We assessed the degree of “local dependence” among scale items by examining item residual correlations. Residual correlations >0.30 warrant further examination,^28^ as they reflect >9% of shared variance.

#### How has the sample been measured? Are responders in the sample separated by the MSIS-29 items?

Scale items are expected to detect differences in levels of impact within a sample and changes over time. Within RMT, the person separation index (PSI) is calculated to assess this.^8,27^ The PSI is a numerical indicator ranging from 0 to 1, computed as the ratio of error-corrected person variance relative to the total person variance,^29^ with higher values indicating greater detection of reliable differences.

#### How valid is the sample measurement?

Similar to item responses, it is important to assess whether the measurement of each person’s total score is in line with RMT expectations.^18^ This is assessed through person fit residual, with reference to the “rule of thumb,” expecting 99% of the sample to produce a fit residual between −2.5 and 2.5. Fit residuals outside this range indicate problematic measurements and questionable measurement validity.^8,27^

#### What is the relationship between MSIS-29 raw scores and interval measurement?

The MSIS-29 total score is ordinal, computed through the summed total of individual Likert-like items rather than an equal-interval measure of Physical or Psychological Impact. It is important to assess the extent to which ordinal raw scores approach interval measurement; one point on an ordinal scale is not necessarily the same across the breadth of the scale,^27,30^ and this has implications when interpreting findings. RUMM2030 plots raw scores against estimated interval measurements, which can be used to provide a subsequent transformation on an interval 0–100 score for each scale.

### Responsiveness analysis

The ability of MSIS-29 scales to detect change at Week 48 was examined and compared. To increase consistency in this comparison, original and restructured scales were anchored on the same overarching scale. Interval level 0–100 transformed scores were used, computed on the basis of RMT-produced interval logit for total raw scores. Responsiveness was examined using four standard indicators: Cohen’s effect size (ES)^31^ and standardized response mean (SRM),^32^ relative efficiency using paired samples *t*-tests,^33^ and relative precision using one-way analysis of variance (ANOVA).^34^

## Results

Data from a total of 1509 people with RRMS at eight time-points are presented in Table 1.

**Table 1.** Sample characteristics at baseline.

	*n*	%
Sex		
Female	1068	10.16
Male	441	29.22
Age		
≤39years	932	61.76
≥40 years	577	38.24
Country region		
USA	1179	78.13
Europe	52	3.45
Rest of the world	278	18.42
Treatment group		
Peginterferon beta-1a 125 mg every two weeks	511	33.86
Peginterferon beta-1a 125 mg every four weeks	500	33.13
Placebo	498	33.00
EDSS score		
1.0 to 2.5 (no to minimal disability)	959	63.55
3.0 to 4.5 (moderate to significant disability)	551	36.51
5.0 to 5.5 (severe disability)	58	3.84

USA: United States of America; EDSS: Expanded Disability Status Scale.

### MSIS-29: RMT findings (Table 2)

**Figure 1.**
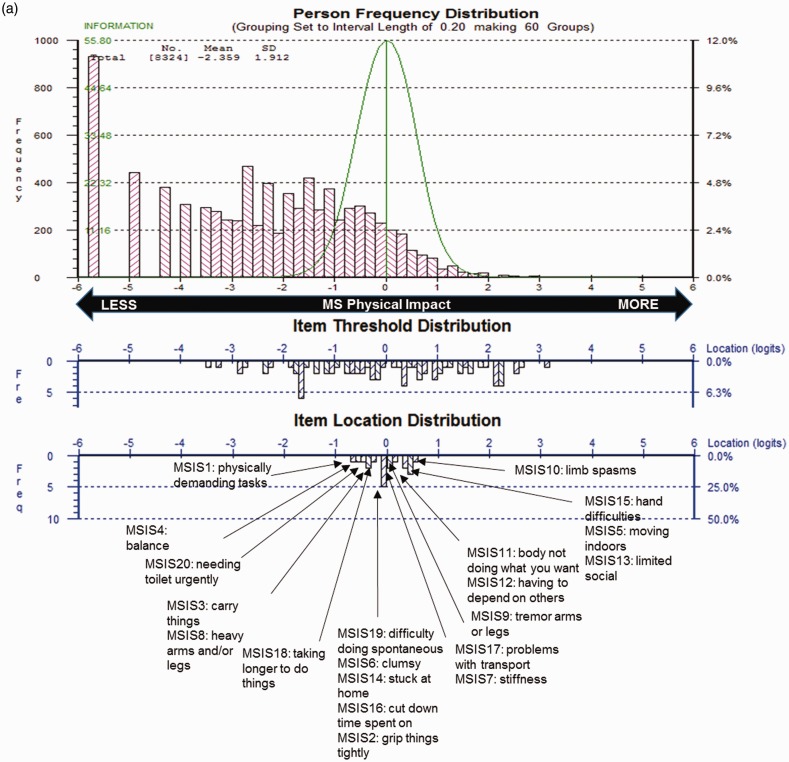

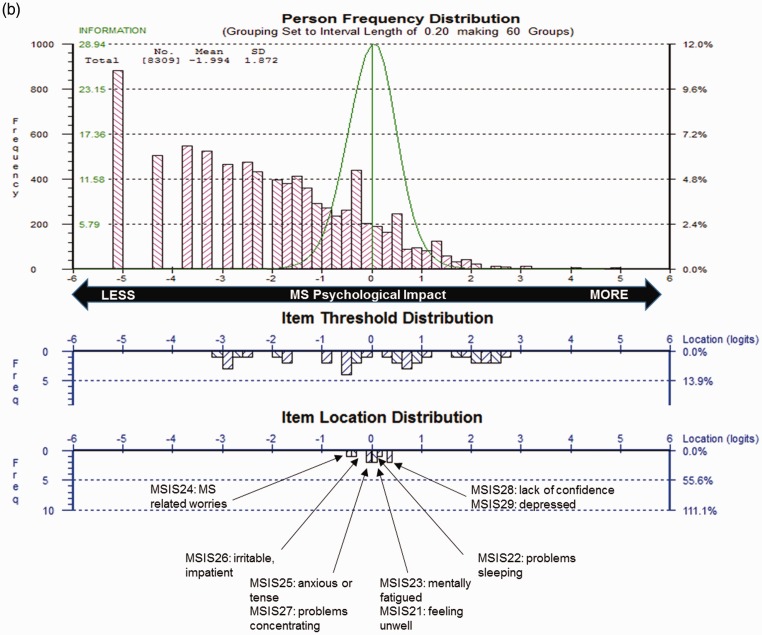
Multiple Sclerosis Impact Scale (MSIS-29) sample-to-scale targeting. The top pink histogram shows the distribution of Physical Impact (a) and Psychological Impact (b) in the sample, and the lower blue histogram shows the distribution of impact in the MSIS-29 scale item thresholds and mean item locations, which map out the 20 (a) and 9 (b) impact items.

MSIS-29 scales demonstrated sub-optimal targeting, as the range of impact measured by scale items covered only 58% of those measured in the sample. Physical Impact (Figure 1(a)) also demonstrated some item bunching, whereas Psychological Impact (Figure 1(b)) had some item gaps on the measurement scale. For both scales, person measurements and means were skewed to the floor; i.e. lower impact.

**Figure 2.**
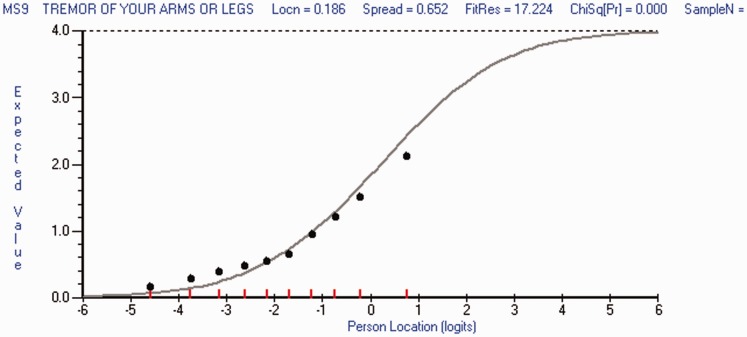
Exemplar item characteristic curve (ICC). The ICC plots the scores expected by the Rasch model for each individual item on the y-axis at each and every level of the measurement continuum of Physical Impact (x-axis). The black dots represent observed scores in each of the 10 class intervals of the trait (i.e. Physical Impact). This ICC for Item 9 indicates slight under-discrimination of the trait, as the line indicated by the dots is flatter than the expected curve. Individuals with higher impact (right hand-side of the continuum) scored lower than expected denoting lower impact, while patients with lower impact (left hand-side of the continuum) scored higher than expected denoting more impact.

All 29 items displayed ordered thresholds, indicating that response categories worked as intended. Item fit residuals for 80% of physical and 89% of psychological items fell outside the recommended range; 15% and 11% failed the adjusted Chi-square criteria, respectively. ICCs reflected marginal fluctuation of observed scores from the Rasch-expected scores, suggesting all four items slightly under-discriminated impact (Figure 2). One item pair had residual correlations >0.30 (*r* = 0.40), suggesting dependency between item responses for “grip things tightly” and “carry things.”

**Figure 3.**
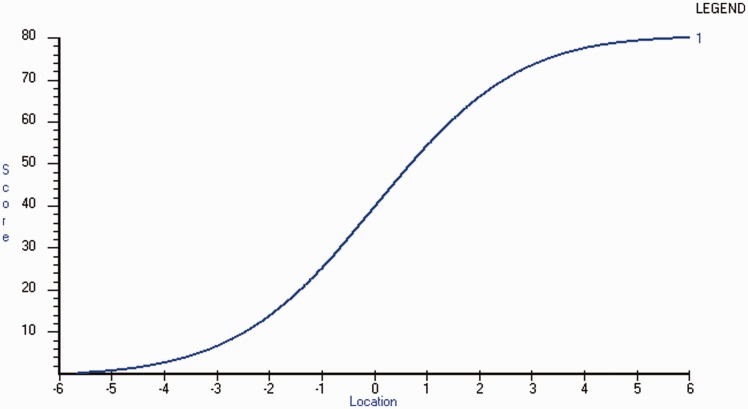
Raw score to interval metric transformation. The x-axis represents the Physical Impact construct as an interval logit score with increasing impact from left to right and the y-axis the raw score as calculated by the summed total of the Multiple Sclerosis Impact Scale (MSIS–29).

PSIs were 0.91 and 0.87, indicating individuals in the sample were separated adequately by MSIS-29 items. Person fit residuals indicated significant misfit for both scales, with 22% of person measurements for Physical and 19% for Psychological Impact falling outside the recommended range, suggesting problematic measurement. The relationship between raw total scores and interval logit metric was S-shaped for both scales, indicating that a one-point change in raw score is associated with a variable rate of change in the impact interval measurement; variability was highest at the two ends and lowest in the center (Figure 3).

### Revised MSIS-29 scoring structure

The MSIS-29 item content was reviewed by a multidisciplinary team that included neurologists, psychologists, and health measurement experts, and three re-conceptualized scales were proposed. The revised scales comprised: “Symptoms,” containing 10 of the original 20 Physical and four of the original Psychological Impact Items; “General Limitations,” containing 10 of the original Physical Impact scales; and “Psychological Impacts,” containing five of its original nine items.

### Revised MSIS-29 scales

**Figure 4.**
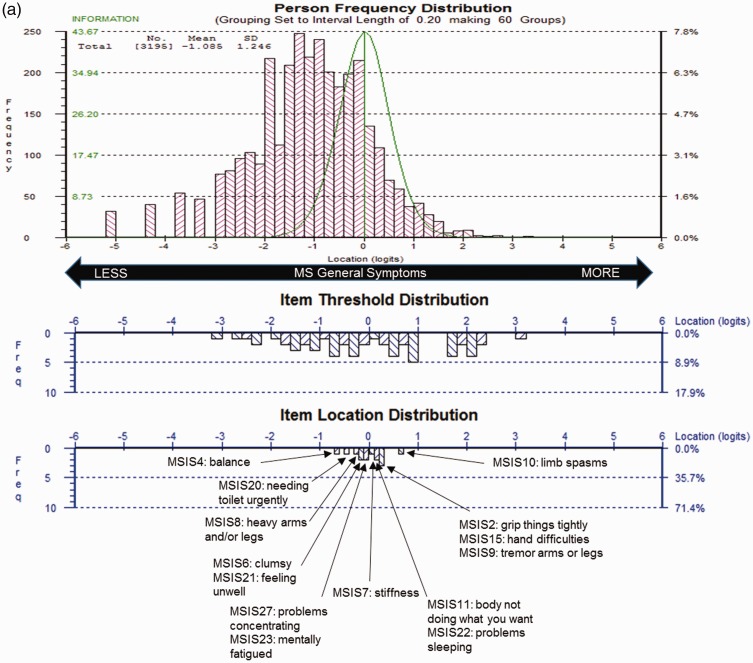

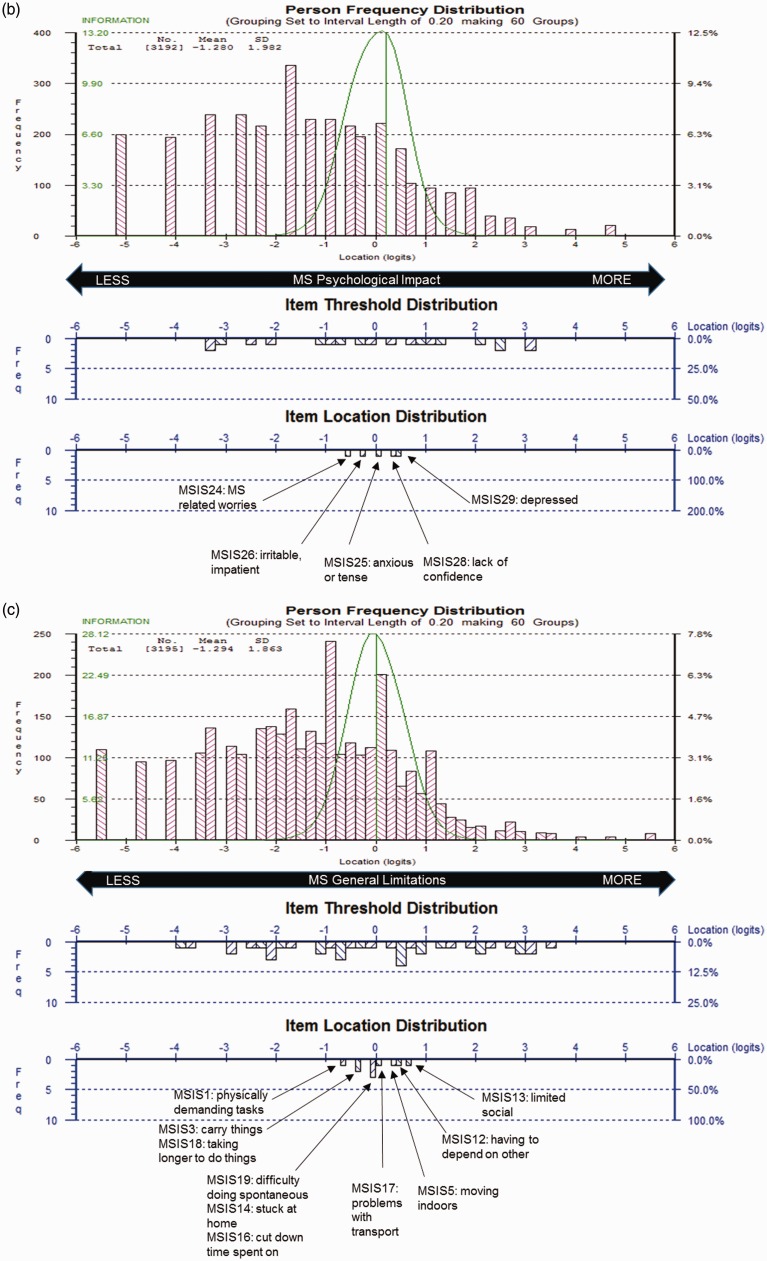
Revised Multiple Sclerosis Impact Scale (MSIS-29) sample-to-scale targeting. The top pink histogram shows the distribution of General Symptoms (a), Psychological Impact (b) and General Limitation (c) in the sample and the lower blue histogram shows the distribution of impact in the MSIS-29 scale item thresholds and mean item locations, which map out the 14 (a), 5 (b) and 10 (c) items.

The revised MSIS-29 scales demonstrated sub-optimal but improved targeting, as the range of impact measured by the scale items covered 68% of the range of the impact measured in the sample, whereas the scales person measurements were consistently skewed to the floor of the measurement scale (i.e. lower impact; Figure 4).

All 29 items displayed ordered item response thresholds. Item fit residuals for 86% of Symptoms, 90% of General Limitations, and 100% of Psychological Impact items fell outside the recommended range, whereas 7% of Symptoms and 10% of Limitations items failed the adjusted Chi-square criteria in the respective scales. ICCs reflected marginal fluctuations of observed scores from the Rasch-expected values, suggesting both items slightly under-discriminated impact. No item dependency was identified.

PSIs ranged between 0.83 and 0.87, indicating adequate separation of individuals by the items. Person fit residuals indicated significant misfit with 16%–28% of person measurements across the scales falling outside the recommended range, suggesting problematic measurement. The relationship between raw total scores and interval logit metric was consistently S-shaped for both scales.

### EDSS sub-sample

Examining sub-optimal targeting primarily at the floor of all scales (i.e. lower impact), scale performance was further assessed on a sub-sample of patients, excluding those with an EDSS score <2.5, reflecting no-to-little disability. Sample-to-scale targeting, the measurement scale, and sample measurement were improved in the original Physical Impact and all three revised scales when tested on the sub-sample of responders with EDSS scores >2.5 (Table 2).

**Table 2.** Overview of RMT findings.

	Targeting distributions		Measurement scale				Sample measurement	
	Person measurements range (mean)	Item threshold range^a^ (%)	Disordered thresholds (%)	Item fit residual range (% outside −2.5/2.5)	Item χ^2^, % significant (*p* < 0.01)^b^	# of item pairs with residual correlations >0.30	PSI	Person fit residual range (% outside −2.5/2.5)
Original								
Physical Impact	–5.68 to 5.53 (–2.36)	3.41 to 3.11 (58%)	0%	–17.30 to 24.72 (80%)	15%	One pair (*r* = 0.40)	0.91	–8.21 to 5.08 (22%, *n* = 1831)
Psychological Impact	–5.18 to 4.86 (–1.99)	–3.17 to 2.66 (58%)	0%	–17.92 to 21.29 (89%)	11%	0 pairs	0.87	–6.54 to 3.88 (19%, *n* = 1549)
Original sub-sample (excluding EDSS ≤2.5 scores)								
Physical Impact	–5.81 to 5.74 (–1.18)	–3.43 to 3.38 (59%)	0%	–10.86 to 17.12 (80%)	10%	One pair (*r* = 0.31)	0.94	–7.97 to 4.92 (19%, *n* = 609)
Psychological Impact	–5.10 to 4.86 (–1.19)	–2.87 to 2.55 (54%)	0%	–10.93 to 14.72 (67%)	11%	0 pairs	0.89	–6.42 to 3.63 (13%, *n* = 423)
Revised								
General Symptoms	–5.01 to 3.29 (–1.96)	–2.64 to 2.97 (68%)	0%	–15.91 to 17.90 (86%)	7%	0 pairs	0.87	–7.07 to 3.86 (16%, *n* = 1343)
Psychological Impact	–5.14 to 4.74 (–2.16)	–3.62 to 3.09 (68%)	0%	–12.96 to 12.41 (100%)	0%	0 pairs	0.83	–3.42 to 3.13 (20%, *n* = 1646)
General Limitations	–5.52 to 5.19 (–2.63)	–4.07 to 3.19 (68%)	0%	–9.96 to 16.63 (90%)	10%	0 pairs	0.87	–5.45 to 4.00 (28%, *n* = 2318)
Revised sub-sample (excluding EDSS ≤2.5 scores)								
General Symptoms	–5.12 to 3.29 (–1.09)	–3.20 to 3.09 (75%)	0%	–7.60 to 10.81 (71%)	0%	0 pairs	0.90	–7.11 to 3.85 (14%, *n* = 441)
Psychological Impact	–5.02 to 4.78 (–1.28)	–3.83 to 3.28 (67%)	0%	–8.37 to 8.13 (60%)	0%	0 pairs	0.86	–3.39 to 3.12 (18%, *n* = 559)
General Limitations	–5.57 to 5.47 (–1.29)	–3.89 to 3.50 (67%)	0%	–7.40 to 12.08 (90%)	0%	0 pairs	0.92	–5.60 to 4.07 (15%, *n* = 468)

RMT: Rasch Measurement Theory; EDSS: Expanded Disability Status Scale; PSI = Person Separation Index; χ^2 ^= Chi-square. ^a^Item threshold mean is always set at 0.00 logits. ^b^Statistical assessment on adjusted sample (*n* = 500) and Bonferroni correction.

### Responsiveness analysis and score interpretation

Relative precision examination suggests that the revised Psychological Impact was the most sensitive scale for detecting change (Table 3). Examining score changes from baseline within the entire sample (Table 4) indicated that only the two Psychological Impact scales (revised and originals) showed a significant (*p* < 0.001) reduction at Week 48, with the revised scale showing the highest relative efficiency. Change scores within the EDSS sub-sample (Table 4) indicated a significant reduction of Psychological Impact in the original and revised scales, and a significant increase in the General Limitations (*p* < 0.01). All three change scores were associated with low ESs and SRMs. Examination of minimally important difference (MID) for ES and SRM compared to mean change scores indicated that they were both higher, providing additional evidence around the magnitude of change scorers for these scales.

**Table 3.** Scale responsiveness: analysis of variance of Rasch transformed 0–100, anchored to scoring algorithm.

	Baseline				Week 48				ANOVA		
	*x*	SD	½ SD	*N*	*x*	SD	½ SD	*F*	*p* value	RP
Placebo whole sample (*n* = 498)
Original Physical Impact^a^	31.00	16.55	8.28	457	30.83	17.35	8.68	0.024	0.878	0.00
Revised Symptoms	31.39	15.32	7.66	457	31.11	16.15	8.08	0.078	0.780	0.01
Original Psychological Impact	33.16	17.97	8.99	457	30.73	18.41	9.21	4.254	0.039	0.60
Revised Psychological Impact	32.82	21.37	10.69	457	29.15	21.10	10.55	7.114	0.008	1.00
Original Physical Impact^b^	29.96	16.49	8.25	457	29.86	17.32	8.66	0.009	0.926	0.00
Revised General Limitation	27.19	19.06	9.53	457	26.99	20.42	10.21	0.023	0.880	0.00
Placebo sub-sample (*n* = 191)
Original Physical Impact^a^	40.15	13.35	6.68	182	40.66	13.64	6.82	0.132	0.717	0.28
Revised Symptoms	39.78	13.24	6.62	182	40.17	13.13	6.56	0.082	0.775	0.17
Original Psychological Impact	39.43	17.56	8.78	182	38.80	16.60	8.30	0.128	0.721	0.27
Revised Psychological Impact	38.93	21.24	10.62	182	37.48	19.28	9.64	0.474	0.491	1.00
Original Physical Impact^b^	40.03	13.41	6.71	182	40.56	13.72	6.86	0.143	0.705	0.30
Revised General Limitation	39.60	15.15	7.57	182	39.12	16.26	8.13	0.088	0.767	0.19
Treatment whole sample (*n* = 1011)
Original Physical Impact^a^	30.98	16.43	8.22	875	29.73	16.69	8.35	0.711	0.399	0.03
Revised Symptoms	30.89	15.29	7.65	874	30.06	15.47	7.74	1.494	0.222	0.07
Original Psychological Impact	32.92	17.12	8.56	873	29.96	18.04	9.21	13.265	0.000	0.60
Revised Psychological Impact	32.54	20.05	10.03	873	28.12	20.68	10.34	22.084	0.000	1.00
Original Physical Impact^b^	29.34	16.43	8.22	875	28.74	16.63	8.32	0.613	0.434	0.03
Revised General Limitation	26.22	19.30	9.65	875	25.92	19.47	9.74	0.112	0.738	0.01
Treatment sub-sample (*n* = 418)
Original Physical Impact^a^	39.34	13.37	6.69	333	39.50	12.89	6.44	0.029	0.865	0.00
Revised Symptoms	39.31	12.61	6.30	333	39.04	12.42	6.21	0.089	0.765	0.01
Original Psychological Impact	39.78	15.69	7.85	333	37.40	16.51	8.25	4.055	0.044	0.62
Revised Psychological Impact	39.37	18.95	9.48	332	35.77	19.38	9.69	6.536	0.011	1.00
Original Physical Impact^b^	39.25	13.45	6.72	333	39.40	12.93	6.47	0.025	0.874	0.00
Revised General Limitation	38.70	16.19	5.40	333	37.30	16.41	8.21	1.361	0.244	0.21

RP: relative measurement precision = (*F*-scale)/(*F*-scale with highest *F* value); ANOVA: analysis of variance. ^a^Original Physical Impact Items anchored on the Physical Impact and General Symptoms merged items scales. ^b^Original Physical Impact Items anchored on the Physical Impact and General Limitations merged items scales.

**Table 4.** Scale responsiveness: paired sample *t*-test of Rasch transformed 0–100, anchored to scoring algorithm.

	Baseline			Week 48											
	Mean	SD	1/2 SD	Mean	SD	1/2 SD	Mean change	SD change	*t*	*p* value	RE	ES	SRM	MID (0.5 ES)	MID (0.5 SRM)
Placebo whole sample (*n* = 455)															
Original Physical Impact^a^	30.66	16.50	8.25	30.76	17.361	8.68	0.11	11.07	0.208	0.836	0.04	0.01	0.01	8.25	5.54
Revised Symptoms	31.17	15.26	7.63	31.04	16.15	8.08	–0.13	10.70	–0.259	0.796	0.05	0.01	0.01	7.63	5.35
Original Psychological Impact	32.99	17.99	9.00	30.62	18.38	9.19	–2.36	13.74	–3.667	0.000	0.78	0.13	0.17	9.00	6.87
Revised Psychological Impact	32.73	21.30	10.65	29.03	21.06	10.53	–3.67	16.71	–4.721	0.000	1.00	0.17	0.22	10.65	8.36
Original Physical Impact^b^	29.62	16.44	8.22	29.8	17.34	8.67	0.18	10.97	0.355	0.723	0.08	0.01	0.02	8.22	5.49
Revised General Limitation	26.65	18.88	9.44	26.92	20.42	10.21	0.27	13.22	0.433	0.665	0.09	0.01	0.02	9.44	6.61
Placebo sub-sample (*n* = 155)															
Original Physical Impact^a^	40.54	12.81	6.41	41.2	13.38	6.69	0.66	10.67	0.768	0.444	0.34	0.05	0.06	6.41	5.34
Revised Symptoms	40.28	12.54	6.27	40.29	12.94	6.47	0.01	10.8	0.010	0.992	0.00	0.00	0.00	6.27	5.40
Original Psychological Impact	40.32	16.87	8.44	38.5	16.56	8.28	–1.81	13.89	–1.625	0.106	0.73	0.11	0.13	8.44	6.95
Revised Psychological Impact	40.15	20.61	10.31	37.46	19.14	9.57	–2.69	16.55	–2.024	0.045	0.91	0.13	0.16	10.31	8.28
Original Physical Impact^b^	40.41	12.85	6.43	41.09	13.46	6.73	0.68	10.73	0.789	0.432	0.35	0.05	0.06	6.43	5.37
Revised General Limitation	40.59	14.73	7.37	40.73	14.68	7.34	0.14	0.757	2.229	0.027	1.00	0.01	0.18	7.37	0.38

	Baseline				Week 48											
	N	Mean	SD	1/2 SD	Mean	SD	1/2 SD	Mean change	SD change	t	p-value	RE	ES	SRM	MID ES	MID SRM
Treatment whole sample																
Original Physical Impact^a^	874	29.75	16.24	8.12	29.76	16.69	8.35	0.01	10.76	0.013	0.990	0.00	0.00	0.00	8.12	5.38
Revised Symptoms	873	30.35	15.22	7.61	30.08	15.46	7.73	–0.26	10.64	–0.732	0.464	0.11	0.02	0.02	7.61	5.32
Original Psychological Impact	869	32.39	17.01	8.51	29.98	18.03	9.02	–2.40	14.22	–4.982	0.000	0.78	0.14	0.17	8.51	7.11
Revised Psychological Impact	869	31.92	19.68	9.84	28.15	20.70	10.35	–3.77	17.37	–6.401	0.000	1.00	0.19	0.22	9.84	8.69
Original Physical Impact^b^	874	28.71	16.22	8.11	28.77	16.63	8.32	0.06	10.64	0.162	0.871	0.03	0.00	0.01	8.11	5.32
Revised General Limitation	874	25.54	18.94	9.47	25.95	19.46	9.73	0.42	12.92	0.951	0.342	0.15	0.02	0.03	9.47	6.46
Treatment sub-sample																
Original Physical Impact^a^	309	39.38	13.04	6.52	39.70	12.84	6.42	0.32	10.08	0.553	0.581	0.16	0.02	0.03	6.52	5.04
Revised Symptoms	309	39.20	12.67	6.34	39.16	12.23	6.12	–0.04	9.81	–0.074	0.941	0.02	0.00	0.00	6.34	4.91
Original Psychological Impact	308	39.63	15.52	7.76	37.39	16.22	8.11	–2.25	14.53	–2.715	0.007	0.78	0.14	0.15	7.76	7.27
Revised Psychological Impact	308	39.30	18.33	9.17	35.72	19.13	9.57	–3.58	17.96	–3.499	0.001	1.00	0.20	0.20	9.17	8.98
Original Physical Impact^b^	309	39.30	13.12	6.56	39.60	12.89	6.45	0.30	10.16	0.520	0.604	0.15	0.02	0.03	6.56	5.08
Revised General Limitation	309	38.13	16.02	8.01	38.21	16.01	8.01	0.07	0.40	3.271	0.001	0.93	0.00	0.19	8.01	0.20

a Original Physical Impact Items anchored on the Physical Impact and General Symptoms merged items scales. ^b^Original Physical Impact Items anchored on the Physical Impact and General Limitations merged items scales. *t*: *t*-statistic; RE: relative efficiency = (*t*-scale)2/(*t*-scale with largest *t*-value)2; ES: effect size; SRM: standardized response mean; MID: minimally important difference.

Table 5 displays the item-level average scores at baseline and Week 48 for both the treatment and placebo groups associated with a range of Rasch-transformed 0–100 scores for the three revised scales. (Results from treatment and placebo groups are displayed together as their results were identical.) Only one item, “feeling depressed,” changed from an average of “a little” to “not at all,” whereas the remaining 28 items had unmoved average responses between the two time-points. Review of these scores indicates scale items are relatively easy for this sample, as 57% and 60% of General Symptoms and Limitations are scored on the floor (“not at all”) on average for both time-points, leaving no room for potential improvement. The remaining items and 80% of the Psychological Impact items are scored on the lower end of impact (“a little”), also leaving limited room for improvement.

**Table 5.** Revised MSIS-29 Scale (Rasch transformed; 0–100): items/response options with associated score ranges for the mean scores at baseline and Week 48.

	0–100 score range for response options:				
	Not at all	A little	Moderately	Quite a bit	Extremely
Symptoms					
Grip things tightly (e.g. turning on taps)	0–37	37–44	44–57	57–63	63–100
Problems with your balance	0–24	24–41	41–51	51–67	67–100
Being clumsy	0–26	26–46	46–59	59–70	70–100
Stiffness	0–32	32–45	45–56	56–74	74–100
Heavy arms and/or legs	0–27	27–41	41–54	54–71	71–100
Tremor of your arms or legs	0–35	35–49	49–57	57–70	70–100
Spasms in your limbs	0–37	37–49	49–59	59–80	80–100
Your body not doing what you want it to do	0–37	37–49	49–59	59–68	68–100
Difficulties using hands in everyday activities	0–38	38–49	49–60	60–69	69–100
Needing to go to the toilet urgently	0–36	36–43	43–48	48–59	59–100
Feeling unwell	0–24	24–44	44–55	55–70	70–100
Problems sleeping	0–35	35–46	46–51	51–63	63–100
Feeling mentally fatigued	0–28	28–45	45–52	52–64	64–100
Problems concentrating	0–26	26–44	44–54	54–65	65–100
Psychological					
Worries related to your MS	0–17	17–40	40–54	54–70	70–100
Feeling anxious or tense	0–18	18–43	43–59	59–80	80–100
Feeling irritable, impatient or short tempered	0–15	15–40	40–56	56–73	73–100
Lack of confidence	0–27	27–47	47–62	62–80	80–100
Feeling depressed	0–30	30–50	50–63	63–76	76–100
Limitations					
Do physically demanding tasks	0–13	13–35	35–54	54–70	70–100
Carry things	0–25	25–39	39–54	54–66	66–100
Difficulties moving about indoors	0–32	32–46	46–63	63–77	77–100
Having to depend on others to do things for you	0–35	35–49	49–59	59–74	74–100
Limitations in your social and leisure activities at home	0–32	32–49	49–61	61–79	79–100
Being stuck at home more than you would like to be	0–31	31–45	45–53	53–69	69–100
Having to cut down time spent on work or other daily activities	0–24	24–44	44–57	57–74	74–100
Problems using transport (e.g. car, bus, train, taxi)	0–36	36–47	47–55	55–66	66–100
Taking longer to do things	0–19	19–42	42–56	56–73	73–100
Difficulty doing things spontaneous (e.g. going out on the spur of the moment)	0–30	30–40	40–55	55–68	68–100

MSIS–29: Multiple Sclerosis Impact Scale; MS: multiple sclerosis. Corresponding Rasch transformed 0–100 score for each response category; responses highlighted in yellow represent the baseline average sample score, in blue Week 48 and in green items for which baseline and week 48 average scores fall within the same response option. Average responses of both the treatment and placebo groups are displayed on same table as they fall within the same response options.

## Discussion

The original MSIS-29 has been an important PRO instrument in MS clinical studies and trials for more than 15 years. Its straightforward scoring for two broad concepts (Physical and Psychological Impact) has allowed for wide application, and provided a strong basis for comparable data between different research endeavors. However, since it was published^3^ there has been an increased use of more sophisticated psychometric methods, which provide the potential for better measurement of patient experience.^1^ Our RMT findings supported previous research,^8^ revealing varied evidence supporting the use of the MSIS-29. In brief, its targeting and conceptual basis could be improved; the range of impact covered by items did not match the range of impact measurement in the study sample (particularly at the floor of the scales, i.e. patients with lower impact), and item fit analyses indicated potential problems for item placement within scales.

We proffer three revised, conceptually clearer scales: “General Symptoms” related to range of symptoms, “Psychological Impact” related to emotional well-being, and “General Limitations” related to difficulties in everyday life. RMT analyses of these revised scales indicated improved sample-to-scale targeting and item fit, although not completely resolved. Our findings also suggest the revised Psychological Impact and General Limitations scales were able to detect more change than original Psychological and Physical Impact scales, respectively. Ultimately, we recommend the MSIS-29 may be further improved by adding more complex (related to higher functioning) items in the lower range of the measurement scale to improve content coverage and floor effects, primarily relevant for patients with mild disability.

Although our findings suggest the potential for improving legacy instruments, such as the MSIS-29, it is important to consider the caveats of our empirically generated revised MSIS-29 scales. First, all post-hoc RMT psychometric analyses of the MSIS-29 are limited to the instrument’s original content, which was not developed in line with RMT, nor were the items selected with clinical hierarchies in mind. Also, considering the original^5^ MSIS-29 was developed with input from patients with relatively high levels of MS disability (>50% were retired because of disability), it is not surprising that the revised scales did not resolve sub-optimal targeting for patients with fewer disabilities.

Using the MSIS-29 to assess clinical change in MS populations similar to the ADVANCE cohort would require an expansion of the scale to include items that are associated with levels of symptoms, psychological impact, and limitations relevant to patients in this context. The proposed scoring structure of the revised MSIS-29, as well as the item hierarchies within each of the revised scales, represent just one way the items could be re-arranged. As this re-conceptualized scoring structure is supported by a single post-hoc psychometric analysis, it is essential that the revised scales be subjected to further psychometric testing and clinical anchoring in independent samples.

Our findings provide an initial evidence base to improve the measurement potential of the MSIS-29 as a PRO instrument in MS clinical research and trials. Articulating scores in relation to symptoms, emotional well-being, and general limitations increases conceptual clarity of MSIS-29. In trials such as ADVANCE, a more explicit and easily interpretable set of concepts can be presented in discussions regarding treatment benefits to patients, regulators, and payers. Additionally, the improved targeting of the revised MSIS-29 scales reduces the overall error associated with measurement. This improves the scales’ potential to reflect the impact of clinical change in MS when it occurs. Finally, since original MSIS-29 scores are ordinal in nature, the use of the linearized (interval-level) transformed 0–100 scoring would benefit the interpretation of scores and change scores, especially in patients with less disability.^35^
